# Role of [^18^F]FDG PET/CT in the management of G1 gastro-entero-pancreatic neuroendocrine tumors

**DOI:** 10.1007/s12020-022-03000-3

**Published:** 2022-02-11

**Authors:** Ludovica Magi, Daniela Prosperi, Giuseppe Lamberti, Matteo Marasco, Valentina Ambrosini, Maria Rinzivillo, Davide Campana, Guido Gentiloni, Bruno Annibale, Alberto Signore, Francesco Panzuto

**Affiliations:** 1 Digestive Disease Unit, Sant’Andrea University Hospital, ENETS Center of Excellence, 00189 Rome, Italy; 2 Department of Anatomical, Histological, Forensic Medicine and Orthopedics Sciences, Sapienza University of Rome, Rome, Italy; 3 Nuclear Medicine Unit, ENETS Center of Excellence, Sant’Andrea University Hospital, 00189 Rome, Italy; 4 Department of Experimental Diagnostic and Specialized Medicine (DIMES), Alma Mater Studiorum, University of Bologna, Bologna, Italy; 5 Division of Medical Oncology, IRCCS Azienda Ospedaliero-Universitaria di Bologna, Bologna, Italy; 6 IRCCS Azienda Ospedaliero-Universitaria di Bologna, Bologna, Italy; 7 Department of Medical-Surgical Sciences and Translational Medicine, Sant’Andrea Hospital, Sapienza University of Rome, Rome, Italy

**Keywords:** Pancreatic endocrine tumors, Prognosis, Management, PET, Grading

## Abstract

**Purpose:**

Since the role of [^18^F]FDG PET/CT in low-grade gastroenteropancreatic (GEP) neuroendocrine neoplasia (NET) is not well established, this study was aimed to evaluate the role of [^18^F]FDG PET/CT in grade 1 (G1) GEP-NETs.

**Methods:**

This is a retrospective study including patients with G1 GEP-NETs who underwent [^18^F]FDG PET/CT.

**Results:**

55 patients were evaluated, including 24 (43.6%) with pancreatic NETs and 31 (56.4%) with gastrointestinal NETs. At the time of diagnosis, 28 (51%) patients had metastatic disease, and 50 (91%) patients were positive by 68-Ga sstr PET/CT. Overall, 27 patients (49%) had positive findings on [^18^F]FDG PET/CT. Following [^18^F]FDG PET/CT, therapeutic management was modified in 29 (52.7%) patients. Progression-free survival was longer in patients with negative [^18^F]FDG PET/CT compared with positive [^18^F]FDG PET/CT (median PFS was not reached and 24 months, respectively, *p* = 0.04). This significance was particularly evident in the pancreatic group (*p* = 0.008).

**Conclusions:**

Despite having low proliferative activity, approximately half of GEP-NETs G1 showed positive [^18^F]FDG PET/CT, with a corresponding negative impact on patients’ clinical outcomes. These data are in favor of a more “open” attitude toward the potential use of [^18^F]FDG PET/CT in the diagnostic work-up of G1 GEP-NETs, which may be used in selected cases to detect those at higher risk for an unfavorable disease course.

## Introduction

Gastroenteropancreatic (GEP) neuroendocrine neoplasia (NEN) is a rare and heterogeneous disease arising from the diffuse neuroendocrine system of the gastrointestinal tract and pancreas [1]. Its prognosis is affected by several factors, including primary tumor site, staging, and grading. The latter is more important for risk stratification and therapeutic choice [2, 3]. According to their morphology and proliferative activity, cases are divided into four categories: NET G1 (well differentiated with Ki67 < 3%), NET G2 (well differentiated with Ki67 3–20%), NET G3 (well-differentiated morphology with Ki67 > 20%), and NEC G3 (poorly differentiated morphology with Ki67 > 20%) [4, 5].

Even if grade 1 (G1) NETs are considered indolent tumors with a very low progressive growth pattern, in some cases, especially in patients with metastases*,* the disease course could be rapidly progressive with a worse response to medical treatment. This reflects an intrinsic hallmark of NENs, tumor heterogeneity, which may translate into nonhomogeneous expression of somatostatin receptors (sstrs) among different tumor lesions at sstr imaging and different Ki67 values between primary and metastatic lesions [6, 7].

Noninvasive functional imaging with positron emission tomography/computed tomography with 18F-fluorodeoxyglucose ([^18^F]FDG PET/CT) has been suggested as a tool for the assessment of NEN aggressiveness and shows prognostic value, particularly in moderate/high-grade (G2 or G3) advanced tumors [8–11]. Conversely, owing to the indolent behavior of G1 NETs, its use in this setting is not advised by international guidelines [12, 13], although positive findings, potentially related to more aggressive tumor behavior and unfavorable patient clinical outcomes, have been reported by some studies [11, 14–16].

Despite its potential clinical utility, data on [^18^F]FDG PET/CT in the management of G1 GEP-NETs are scarce and are mainly derived from heterogeneous populations of NENs, including a few G1 tumors.

Thus, in this study, we aimed to evaluate the utility of [^18^F]FDG PET/CT in the specific setting of well-differentiated G1 GEP-NETs.

## Materials and methods

### Study design

This was a retrospective study including all consecutive patients with a histologically proven diagnosis of G1 GEP-NETs managed by multidisciplinary teams (MDTs) in two Italian European Neuroendocrine Tumor Society (ENETS) Centers of Excellence (Sant’Andrea University Hospital Center, Rome, and IRCCS Azienda Ospedaliero-Universitaria di Bologna) who underwent [^18^F]FDG PET/CT. The study was carried out in accordance with the Declaration of Helsinki, and full informed consent for data collection was obtained from all patients.

In accordance with the centers’ standard procedures and following the ENETS guidelines, all major clinical and pathological data were collected in an anonymized database.

The diagnosis of GEP-NETs was based on conventional histological findings [17]; tumor grade was established according to the World Health Organization (WHO) 2019 classification [3, 4], and disease stage was assessed according to the ENETS TNM staging system [18, 19]. All patients were discussed by MDT, which is active in each center. If required, after MDT discussion, new tumor biopsy sampling was performed in those cases with unclear diagnoses.

A follow-up duration of at least 6 months after [^18^F]FDG PET/CT evaluation was also required to include the patient in the final analysis. Patients were divided into two groups according to the indication for performing [^18^F]FDG PET/CT: i. assessment of disease aggressiveness at the time of initial NET diagnosis or ii. evaluation of tumor behavior at the time of radiologically documented progression of disease during follow-up. Although not standardized, the decision to perform [^18^F]FDG PET/CT was made after the center MDT had discussed the case [20]. The ability of [^18^F]FDG PET/CT to change clinical management was defined as the occurrence of one of the following conditions as a consequence of the MDT discussion due only to the [^18^F]FDG PET/CT finding: i. start new therapy; ii. repeat bioptic sampling; iii. plan surgical procedure. Data were retrieved from the MDT activity reports at each center. In those patients for whom an initial NEN diagnosis was made before referring to the center, a pathological revision was performed by an expert pathologist in NENs, according to the ENETS standard of care [21]. Patients in whom the primary tumor site was unknown were also included if the tumor was believed to belong to the small bowel according to histological examination (synaptophysin+; CDX2+, serotonin+) and after other common primary sites were ruled out by conventional imaging procedures (CT or MRI, as appropriate, and 68-Ga sstr PET/CT).

Follow-up was performed by both participating centers according to the ENETS standard of care [21] by CT or MRI every 3–6 months depending on the clinical scenario.

During follow-up, the disease status was assessed according to Response Evaluation Criteria in Solid Tumors version 1.1 criteria [22].

### Imaging protocol

[^18^F]FDG PET/CT examinations were performed in both centers using a hybrid PET/CT system (Biograph Horizon, Siemens, Germany [at the Sant’Andrea University Hospital Center, Rome] and GE discovery MI, GE discovery STE, GE discovery 710 [at the S. Orsola-Malpighi University Hospital, Bologna]). In each center, the [^18^F]FDG PET/CT examination was acquired according to the European Association of Nuclear Medicine (EANM) Guidelines [23]. A positive [^18^F]FDG PET/CT finding was defined as the presence of at least one abnormal area of focal FDG uptake outside the physiological distribution or with higher uptake than the surrounding physiological tissue.

### Statistical analysis

The distribution of continuous variables was reported as the median and interquartile range (IQR; 25th–75th percentiles) or range, as appropriate. Progression-free survival (PFS) was defined as the interval between [^18^F]FDG PET/CT examination and the time of PD or patient death if it occurred before documented PD. PFS and overall survival (OS) analyses were performed using the Kaplan–Meier method, and the results were compared by the log-rank test. Risk factor analysis to identify clinical variables associated with an increased risk of progression was performed by Cox proportional hazards regression. The *P* value was considered significant when it was < 0.05. Statistical analysis was performed by MedCalc^®^ v.17 software (MedCalc Software, www.medcalc.org).

## Results

### Included patients

A total of 80 patients were evaluated for potential inclusion in the study. Of these, 25 (31.2%) patients were excluded because minimal follow-up data were not available (*n* = 10), there was a grading modification after repeating histological assessment in patients referred to the Centers from other hospitals (*n* = 10), and a Ki67 value was missing and it was not possible to repeat histological assessment due to tissue unavailability (*n* = 5). Thus, the final analysis was performed in 55 patients (Table 1), including 36 males (65.4%) with a median age of 63 (IQR 25–83) years. Thirty-one patients had gastrointestinal (GI) primary NETs (56.4%), including 26 ileal, 1 rectal, 2 duodenal, 1 colonic, and 1 gastric NET, and 24 patients (43.6%) had pancreas as the primary site. At the time of diagnosis, 28 patients (51%) had stage IV disease with metastases predominantly found in the liver (85.5%); 10 (18.2%) had lymph node metastases, and the other 2 (3.6%) patients had bone metastases. The most frequent indication for [^18^F]FDG PET/CT examination was assessment of disease aggressiveness at initial NET diagnosis in 38 (69%) patients, including patients with negative 68-Ga sstr PET/CT (10.5%) or inhomogeneous 68-Ga sstr PET/CT uptake (7.9%), patients with multiple comorbidities suitable for surgery in whom [^18^F]FDG PET/CT was required before operation (50%), patients with extensive tumor burden (15.8%), and finally patients (15.8%) with nonfunctioning symptomatic disease (i.e., pain, weight loss). Otherwise, 17 patients (31%) underwent [^18^F]FDG PET/CT due to evidence of disease progression, which occurred after a median interval from initial diagnosis of 30 (IQR 2–180) months. Overall, [^18^F]FDG PET/CT examination was acquired after a median frame of 5 months (IQR 0–180) from the histological diagnosis.

**Table 1:** Characteristics of the study population

Characteristics	Overall (*n* = 55) *n* (%)	[^18^F]FDG PET/CT + (*n* = 27) *n* (%)	[^18^F]FDG PET/CT − (*n* = 28) *n* (%)	*p* value
Primary tumor site				
Pancreas	24 (43.6%)	13 (54.2%)	11 (45.8%)	0.5
Gastrointestinal	31 (56.4%)	14 (45.2%)	17 (54.8%)	
Tumor staging				
Stage I–II	13 (23.6%)	3 (23.1%)	10 (76.9%)	0.05
Stage III–IV	42 (76.3%)	24 (57.1%)	18 (42.9%)	
Metastases site				
Liver	24 (43.6%)	11 (45.8%)	13 (54.2%)	0.15
Extra- hepatic	12 (21.8%)	9 (75%)	3 (25%)	
^68^Ga PET positive	50 (91%)	25 (50%)	25 (50%)	1
Previous treatment				
Yes	19 (34.5%)	9 (47.4%)	10 (52.6%)	1
No	36 (65.4%)	18 (50%)	18 (50%)	

*[*^*18*^*F]FDG PET/CT* positron emission tomography/computed tomography with 18F-fluorodeoxyglucose, ^*68*^*Ga PET*, 68-Ga somatostatin receptor PET/CT

### [^18^F]FDG PET/CT findings

Overall, 27 (49%) patients had a positive [^18^F]FDG PET/CT. The characteristics of the FDG-positive population are summarized in Table 1. Of these, at the time of [^18^F]FDG PET/CT examination, 18 patients (66.7%) were not receiving any medical antitumor treatment, whereas 9 patients (33.3%) were receiving systemic therapy.

After [^18^F]FDG PET/CT, a change in clinical management was proposed in 29 (52.7%) patients.

**Fig. 1:**
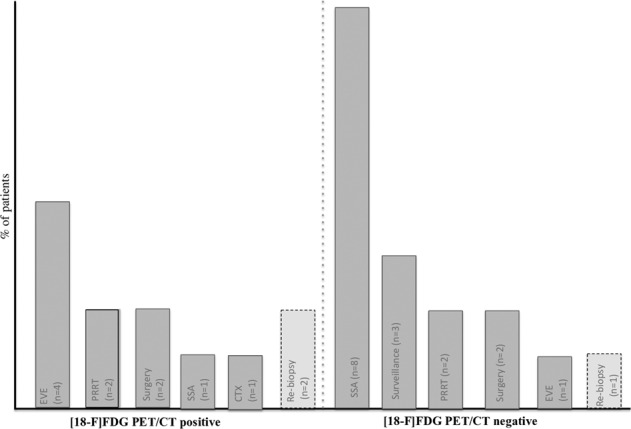
MDT decision after [^18^F]FDG PET/CT findings. Abbreviations: MDT, multidisciplinary Team; [^18^F]FDG PET/CT, 18F-fluorodeoxyglucose positron emission tomography/computed tomography; EVE Everolimus, PRRT peptide receptor radionuclide therapy, SSA somatostatin analogs, CTX chemotherapy

Of these, 12 (41.4%) patients had positive findings on [^18^F]FDG PET/CT, whereas 17 (58.6%) patients had negative examinations. The most frequent medical treatment that patients received after negative [^18^F]FDG PET/CT was somatostatin analogs (SSAs) in 8 (47%) patients, whereas more aggressive management was carried out in those with positive [^18^F]FDG PET/CT. In 3 (5.4%) patients, rebiopsy was performed to rule out potential changes in tumor biology; of these, 1 (2%) patient, with a positive [^18^F]FDG PET/CT evaluation, showed a change in tumor grade that moved from G1 (Ki67 1%) to G3 grading (Ki67 30%). Decisions on patient management taken by the center MDT after [^18^F]FDG PET/CT findings are reported in Fig. 1.

### Patient follow-up after FDG PET

**Fig. 2:**
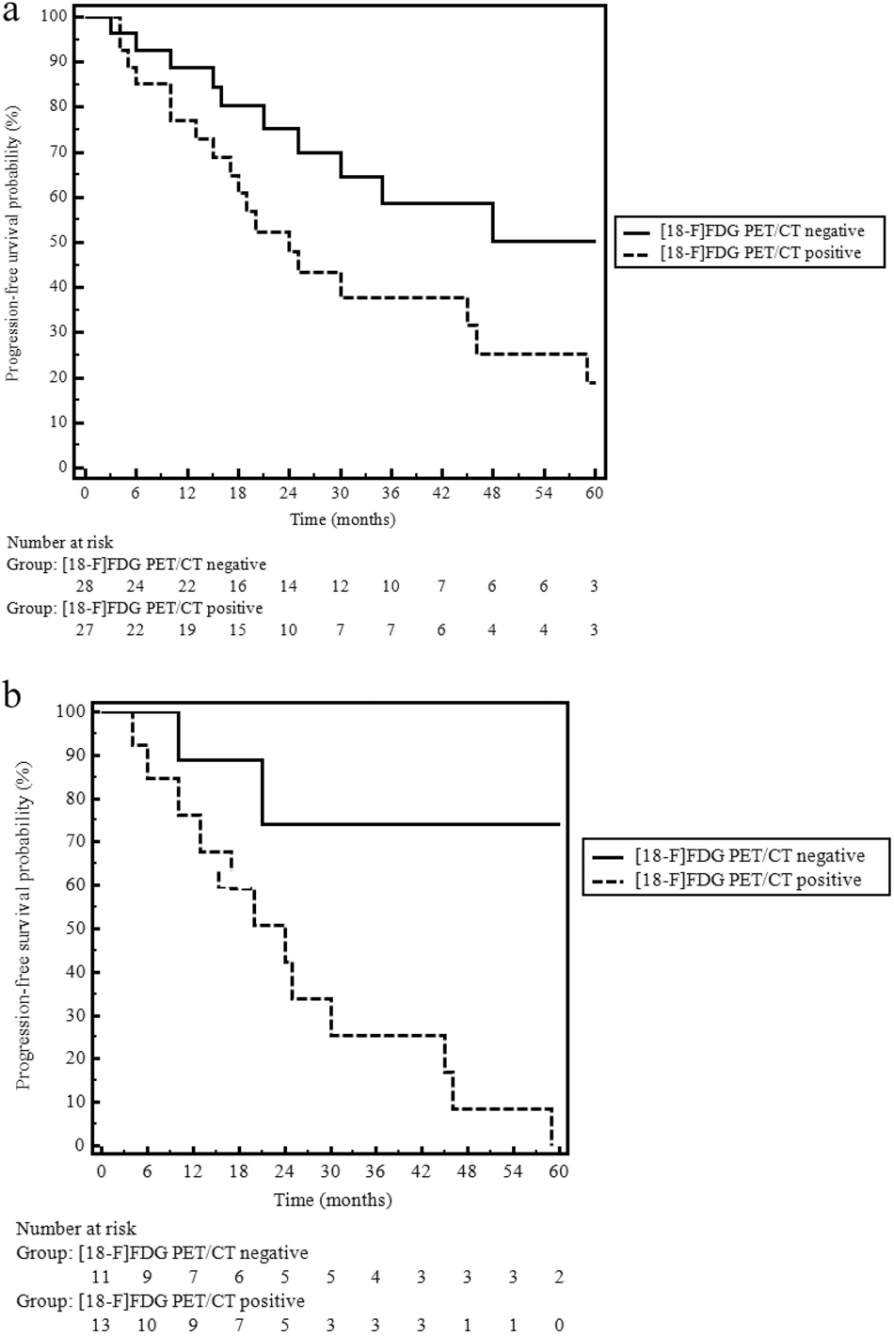
**a** Progression-free survival according to [^18^F]FDG PET /CT. Abbreviations: [^18^F]FDG PET /CT 18F-fluorodeoxyglucose positron emission tomography/computed tomography; **b** Progression-free survival according to [^18^F]FDG PET /CT findings in the pancreatic group. Abbreviations: [^18^F]FDG PET /CT 18F-fluorodeoxyglucose positron emission tomography/computed tomography

Overall, 29 (52.7%) patients had PD on cross-sectional radiological imaging performed during follow-up after [^18^F]FDG PET/CT with a median PFS of 38 months; of these, 14 (48.3%) had the pancreas as the primary site, whereas 15 (51.7%) had other gastrointestinal origins. PFS was significantly longer in patients with negative [^18^F]FDG PET/CT than in those with positive [^18^F]FDG PET/CT, and the median PFS was “not reached” and 24 months (*p* = 0.04; Fig. 2a) in the two subgroups, respectively. This significance was particularly evident in the pancreatic group (*p* = 0.008; Fig. 2b), whereas no significance was observed in the GI group.

Analysis to identify other potential predictors for tumor progression showed that no factor was associated with poor clinical outcome, including pancreatic origin; the only significant predictor was the [^18^F]FDG PET/CT finding (Table 2). A total of 14 patients died during follow-up, resulting in a mortality rate of 25.4%. The median OS times were 31 months in the general population and 27 months in the pancreatic group. No significant difference in terms of survival was observed in patients with positive or negative [^18^F]FDG PET/CT, either in pancreatic or GI primary tumor sites.

**Table 2:** Predictor variables associated with tumor progression

Variable	HR	95% CI	*p* value
Treatment before [^18^F]FDG PET/CT (yes vs no)	1.66	0.78–3.49	0.183
Timing of [^18^F]FDG PET/CT (diagnosis vs progression)	1.69	0.81–3.54	0.163
Primary tumor site (pancreas vs GI)	1.34	0.64–2.77	0.43
Metastatic (Yes vs No)	1.87	0.87–4.01	0.108
Gender (male vs female)	1.59	0.72–3.51	0.245
^68^Ga-PET finding (positive vs negative)	0.94	0.22–3.99	0.933
[^18^F]FDG PET/CT (positive vs negative)	2.17	1.01–4.69	0.04

*[*^*18*^*F]FDG PET/CT* positron emission tomography/computed tomography with 18F-fluorodeoxyglucose; ^*68*^*Ga PET* 68-Ga somatostatin receptor PET/CT

## Discussion

To date, the clinical role of [^18^F]FDG PET/CT in the management of well-differentiated G1 GEP NETs still needs to be established. To address this issue, the present study reports data on the use of [^18^F]FDG PET/CT in a homogeneous population of well-differentiated, “indolent”, G1 GEP NETs to understand whether this examination may help physicians to better predict tumor behavior and patient clinical outcomes.

**Fig. 3:**
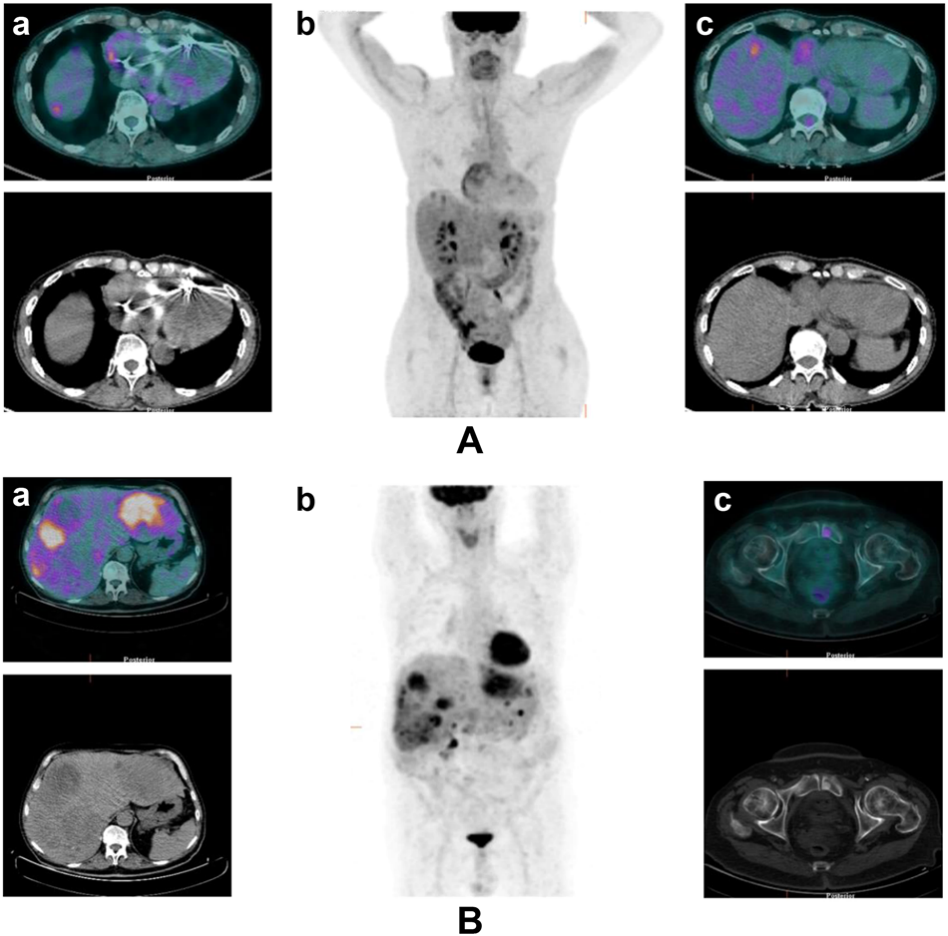
Two cases of patients with positive [^18^F]FDG PET /CT. **A** A 78-year-old female patient with newly diagnosed ileal NET G1 with liver metastases. (a, c) Axial FDG- PET/CT and (b) maximum intensity projection (MIP) images showing pathological uptake in two liver lesions. **B** A 73-year-old-male patient affected by pancreatic NET G1 with liver, lymph node, and bone metastases. (a) Axial [^18^F]FDG PET/CT and (b) maximum intensity projection (MIP) images showing pathological uptake in several liver lesions and in small bone lesion (c). Abbreviation: [^18^F]FDG PET/CT fluorodeoxyglucose positron emission tomography/computed tomography

The first interesting finding involves the fact that, notwithstanding that it is not recommended by current guidelines, a significant proportion of patients (49%) had positive [^18^F]FDG PET/CT findings (an example of [^18^F]FDG PET/CT-positive findings is shown in Fig. 3). Similar to what is already known in more aggressive NENs, the present study shows that positive [^18^F]FDG PET/CT is associated with a poor clinical outcome in terms of progression-free survival (PFS not reached in [^18^F]FDG PET/CT-negative patients and 24 months in FDG-positive patients, *p* = 0.04).

This figure, which may sound unexpected owing to the low proliferative activity of these tumors, might be related to several factors. First, it is consistent with the observation that [^18^F]FDG PET/CT positivity in GEP NET lesions is not related only to grading but also depends on tumor behavior, growth rate, and glucose transporter expression [8]. Although published data are usually extracted from heterogeneous populations, including NENs of any grade, other papers have reported [^18^F]FDG PET/CT positivity even in low-grade tumors, suggesting its potential utility in their clinical management [10]. Moreover, it has been suggested that [^18^F]FDG PET/CT could be superior to histological grading for risk stratification of all NEN grades, particularly to differentiate G1 and G2 into low- and high-risk groups [24]. Although previous studies reported that [^18^F]FDG PET/CT may have a prognostic role even in low-grade NENs [9, 11], analyzing data on the specific setting of G1 NETs is usually not feasible, given the heterogeneity of the populations included and the low proportion of G1 tumors analyzed.

Another important finding reported in the present study is that after [^18^F]FDG PET/CT, a change in clinical management was proposed in 52.7% of patients. In general, MDT decisions after negative [^18^F]FDG PET/CT involved treating patients with somatostatin analogs (47%), following patients with active surveillance without administering therapy (17.6%), or planning PRRT (11.7%). Conversely, after evaluating a positive [^18^F]FDG PET/CT finding, a significant proportion of patients received everolimus (33.3%) or PRRT (16.6%); interestingly, MDT decided to repeat biopsy to obtain a new grading assessment in 2 (16.6%) cases.

Although the impact of 68-Ga sstr PET/CT on the clinical management of NET patients is well known [25, 26], the potential role of [^18^F]FDG PET/CT in this particular clinical scenario is not well established. Conversely, to the results of the present study, Panagiotidis et al. [27] reported that [^18^F]FDG PET/CT has no clinical impact on G1 NETs and only a moderate impact on the management of G2 NETs. In fact, in that study, the clinical management was changed based on [^18^F]FDG PET/CT in only 1 out of 36 G1 NET patients; however, when considering the role of [^18^F]FDG PET/CT alone or combined with 68-Ga sstr PET/CT, the clinical management was modified in 11 out of 36 patients (30.5%), suggesting that [^18^F]FDG PET/CT might play a role in making decisions, although 68-Ga sstr PET/CT remains predominant [28]. However, a direct comparison with that study is not feasible, owing to the differences concerning the study endpoints and the population enrolled (in that study, mixed primary tumors often with recurrent disease were included).

Although the current guidelines [29] recommend [^18^F]FDG PET/CT examination only for G3 NENs, the present study suggests that [^18^F]FDG PET/CT may also have a role in the management of low-grade G1 NETs. In general, since [^18^F]FDG PET/CT positivity usually correlates with poor clinical outcome [11, 24], a negative finding may help clinicians choose less aggressive therapeutic approaches, whereas more aggressive management could be advised in [^18^F]FDG PET/CT-positive patients, irrespective of the tumor grading. This figure is also confirmed by the present study, which reports a significantly poorer PFS in [^18^F]FDG PET/CT-positive patients than in [^18^F]FDG PET/CT-negative patients, particularly in those with tumors of pancreatic origin. This figure confirms what is already known about the more aggressive behavior of NETs with a pancreatic origin, compared with GI primaries [30, 31].

Although tumor grade remains the most powerful prognostic factor in NENs [2, 32, 33], it has the limitation of being obtained by random biopsy, potentially reflecting only a limited part of the tumor. Performing [^18^F]FDG PET/CT may help to overcome this potential limitation by providing complementary information regarding the metabolic activity of the whole disease.

Based on the major findings of the present study, we suggest that [^18^F]FDG PET/CT may be considered in the work-up of G1 GEP-NETs, particularly for those with a pancreatic origin, owing to the possibility of obtaining a positive finding, which might be helpful for patient clinical management and to predict clinical outcome. Compared with the existing data, this study provides additional knowledge concerning the potential role of [^18^F]FDG PET/CT in low-grade G1 GEP-NETs [34]. However, we are aware that this study has major limitations due to its retrospective design and the relatively low number of patients included, which are intrinsic weaknesses of most studies including NENs related to the rarity of this disease. Additional limitations are the time frame during which [^18^F]FDG PET/CT was performed (median of 5 months from histological diagnosis) and the absence of a standardized protocol suggesting how to select patients in whom this examination was performed since this was based on MDT choice in each given patient.

## Conclusion

Although [^18^F]FDG PET/CT has an emerging role in defining tumor aggressiveness and predicting clinical outcome in high-grade NENs, its role in well-differentiated G1 NETs is not well established. The present study shows that despite having extremely low proliferative activity, approximately half of GEP-NETs G1 show a positive finding at [^18^F]FDG PET/CT, which correlates with a poor patient clinical outcome, particularly in those with tumors of pancreatic origin. After performing [^18^F]FDG PET/CT, changes in clinical management were made in a significant proportion of patients. These data are in favor of a more “open” attitude toward the potential use of [^18^F]FDG PET/CT in the diagnostic work-up of G1 GEP-NETs, which may be useful to select patients at higher risk for unfavorable disease courses.
